# Interleukins, laminin and epstein - barr virus latent membrane protein 1 (EBV LMP1) Promote metastatic phenotype in nasopharyngeal carcinoma

**DOI:** 10.1186/1471-2407-10-574

**Published:** 2010-10-22

**Authors:** Michelle MS Chew, Sook-Yee Gan, Alan SB Khoo, Eng-Lai Tan

**Affiliations:** 1 Department of Research and Postgraduate Studies, International Medical University, No. 126, Jalan 19/155B, Bukit Jalil, Kuala Lumpur 57000, Malaysia; 2 Department of Pharmacy and Health Sciences, International Medical University, No. 126, Jalan 19/155B, Bukit Jalil, Kuala Lumpur 57000, Malaysia; 3 Cancer Research Centre, Institute for Medical Research, Jalan Pahang, 50588 Kuala Lumpur, Malaysia

## Abstract

**Background:**

Nasopharyngeal carcinoma (NPC) is a type of neoplasm that is highly prevalent in East Asia and Africa with Epstein-Barr virus (EBV), genetic, and dietary factors implicated as possible aetiologic factors. Previous studies suggested the association of certain cytokines with the invasion and metastatic properties of NPC. The present study examined the roles of EBV latent membrane protein-1 (LMP1), interleukin-6 (IL-6), interleukin-10 (IL-10), transforming growth factor-beta 1 (TGF-β1) and laminin in the regulation of matrix-metalloproteinases (MMPs) and vascular endothelial growth factor (VEGF) in NPC. The effects of these factors on *bmi-1*, an oncogene, and *ngx6*, a tumour suppressor gene, were also investigated.

**Methods:**

TW01 cells expressing LMP1 (TW01-LMP1) were established via transfection with the B95.8 EBV LMP1 gene. Both TW01 and TW01-LMP1 cells were treated with 100 pg/ml IL-6, 1000 pg/ml IL-10 and 100 pg/ml TGF-β1, separately and also in combination at their respective concentration for 48 hours. Treated cells were subjected to laminin adherence assay. The cells were also cultured with and without laminin and assayed for MMP-3, MMP-9 and VEGF production using enzyme-linked immunosorbent assay (ELISA). The cellular apoptotic property was analysed using caspase-3 apoptosis assay. The expression of *bmi-1 *and *ngx6 *gene was investigated using real time reverse transcriptase polymerase chain reaction.

**Results:**

LMP1 was found to reduce the adherence of NPC cells towards laminin (p < 0.05) as compared to control. Treatment with IL-6 at 100 pg/ml enhanced the production of MMP-9 in both TW01 and TW01-LMP1 cells (p < 0.05). When cultured on laminin, the levels of MMP-3 and VEGF were significantly increased (p < 0.05) in TW01-LMP1 cells. TW01-LMP1 cells had relatively greater resistance to apoptosis as compared to TW01 cells (p < 0.05). Laminin, IL-6 and LMP1 were found to up-regulate the expression of *bmi-1 *and suppressed the expression of *ngx6*.

**Conclusions:**

We conclude that IL-6 reduced cell adherence towards laminin and increased MMP-9 production in NPC cells. Our data suggested that EBV LMP1 was able to confer resistance of apoptosis and increased MMP-9 production in NPC cells. When cultured on laminin, TW01 cells expressing the EBV LMP1 (TW0-LMP1) that were treated with IL-6 at 100 pg/ml displayed increased MMP-9 production, up-regulation of *bmi-1 *oncogene expression and down-regulation of *ngx6 *tumour suppressor gene expression. These findings implicate the roles of EBV LMP1, laminin and IL-6 in the promotion of invasion and metastasis in NPC.

## Background

Nasopharyngeal carcinoma (NPC) is a disease with an extraordinary geographic and racial distribution worldwide. Except for a handful of populations, NPC is a rare human malignancy with an incidence well under 1 per 100 000 population per year, constituting less than 0.3% of all malignant tumours and only 2% of all head and neck cancer [1]. The most suspected etiologic factors of NPC are genetic susceptibility, infection with Epstein-Barr virus (EBV), and regular consumption of salted fish beginning in childhood [2].

EBV is an ubiquitous human gamma-herpes virus that is commonly associated with a number of malignancies such as Burkitt's lymphoma, Hodgkin's disease, stomach carcinomas and NPC [3]. NPC patients have elevated IgG and IgA antibody titers to the EBV viral capsid antigen (VCA) and to antigen associated with replication, called early antigen (EA) [4]. Elevated expression of EBV latent membrane protein-1 (LMP1) is correlated with tumour progression and metastasis [5].

Until now, the treatment of cancer metastasis, including NPC, still remain as the greatest obstacle. Laminin is the main non-collagenous glycoprotein found in the basement membrane. The interaction of cancer cells with laminin was acknowledged as a key event in tumour invasion and metastasis [6]. In tumours, laminin is produced by tumour cells and also in the extracellular matrix (ECM) [6]. According to Lee *et al.*, LMP1 promotes metastasis in NPC by inducing matrix metalloproteinases (MMPs) in degrading ECM proteins [5]. MMPs have the ability to digest a broad range of ECM molecules. These enzymes have been implicated in the turnover of the ECM during tumour development and progression [7]. MMP-3 expression is a prognostic indicator of invasion and lymph node metastasis in head-and-neck squamous cell carcinoma [8]. Subsequent study showed that a significant level of MMP-3 was detected in NPC patients compared with other head-and-neck cancer patients [9]. The expression of MMP-9 is positively correlated with the expression of LMP1, as well as with the metastasis of NPC in patients [3,10].

Vascular endothelial growth factor (VEGF) is another important cytokine that plays an important role in endothelial cell proliferation and the process of angiogenesis which are essential for tumour development [7]. In addition, Khrishna *et al*. has found that expression pattern of VEGF could be used as a potential tumour marker for the early diagnosis of NPC metastasis and they showed that the upregulation of VEGF is associated with the presence of EBV [11].

The link between inflammation and cancer has long been recognised about 150 years ago [12,13]. It is now becoming clear that the tumour microenvironment, which is largely surrounded by inflammatory cells, is a crucial participant in the neoplastic process, fostering proliferation, survival and migration [13]. It was reported that serum level of interleukin-6 (IL-6) was elevated in NPC and prostate cancer patients [14,15]. Interestingly, interleukin-10 (IL-10) and transforming growth factor-beta1 (TGF-β1) have been regarded as cytokines that serve dual roles in cancer progression. They exert anti-carcinogenic functions but play vital roles in tumour progression [16,17]. However, the roles of IL-6, IL-10 and TGF-β1 on the invasion and metastasis on NPC are still unclear.

The present study compares the effects of IL-6, IL-10, TGF-β1 and laminin on the biological properties of NPC TW01 cells with and without EBV LMP1 expression. The roles of EBV LMP1, IL-6, IL-10, TGF-β1 and laminin in the regulation of MMPs and VEGF in NPC were examined. The effects of these factors on *bmi-1*, an oncogene, and *ngx6*, a tumour suppressor gene, were also investigated.

## Methods

### Cell Lines

B95.8 cell is a lymphoblast-like cell line derived from Marmoset blood lymphocytes which were exposed to EBV from human leukocyte line. This cell line was cultured in DMEM:F12 (Invitrogen, USA) supplemented with 10% v/v fetal bovine serum (Invitrogen, USA) and maintained in a 37°C, 5% CO_2 _incubator. TW01, a human NPC cell line obtained from a 64-year old male Taiwanese patient by Lin *et al.*, was maintained using the same procedure [18]. Both the TW01 and TW01-LMP1 cell lines have been characterised by Tan *et al*. in their previous study [19].

### Cloning of LMP1 gene

EBV RNA was obtained from B95.8 cells using RNeasy Mini kit (Qiagen, USA). Reverse transcription (RT) followed by Polymerase Chain Reaction (PCR) was performed using Superscript™ III One-Step RT-PCR System (Invitrogen, USA) according to the manufacturer's protocol. LMP1 gene specific primers with the forward LMP1 sequence of 5'- CACCATGGATGGAACACGACCTTGAG -3' and reverse LMP1 sequence of 5'-GACAGTGTGGCTAAGGGA-3' were used at 0.2 μM in 50 μl master mix containing 1X Reaction Mix (Invitrogen, USA), 1 unit RT/Platinum mix (Invitrogen, USA), 15 μl sterile water and 1 μg of template RNA. RT was performed at 50°C for 30 min and terminated by incubation at 94°C for 2 min. Amplification was performed using the following parameter; 94°C for 15s; 53°C for 30s and 68°C for 1 min with the final extension of 68°C for 5 min. Amplified products were analysed by agarose gel electrophoresis and fragments of the correct size (1373 base pairs) were cloned into the pcDNA3.1 Directional TOPO vector (Invitrogen, USA) according to the manufacturer's recommendation. The vector was transformed into *Escherichia coli *for multiplication. Proper integration of LMP1 gene into pcDNA3.1 Directional TOPO vector was confirmed by DNA sequencing (Research Biolabs, Singapore).

### Transfection of LMP1 gene into TW01 cells

LMP1-expressing TW01 cell clones were established by transfecting the cells with pcDNA-LMP1 plasmids that carried recombinant LMP1 gene derived from B95.8 cells. Transfection of the epithelial cells was performed using Lipofectamine™ 2000 (Invitrogen, USA) as recommended by the manufacturer. Transfected cells were incubated for 24 h at 37°C with 5% CO_2 _and transgene expressing cells were selected by passaging at 1:10 dilutions in fresh selection of medium, DMEM-F12 supplemented with 230 μg/ml Geneticin (G418). The LMP1 protein expression was further confirmed by Western blotting with monoclonal mouse anti-EBV LMP1 CS. 1-4 (Dako, Denmark). TW01 expressing the LMP1 gene is designated as TW01-LMP1. Non transfected TW01 cell line was used as the negative reference for Western blotting. The expression of LMP1 in the transfected TW01 cells was also confirmed with RT-PCR using the same procedure mentioned above.

### Laminin Adhesion Assay

The relative cell attachment to laminin was assessed using Innocyte™ ECM Cell Adhesion Assay, Laminin/Basement Membrane Complex (Calbiochem, USA). Prior to the assay, TW01 and TW01-LMP1 expressing cells were treated with 100 pg/ml IL-6, 1000 pg/ml IL-10 and 100 pg/ml TGF-β1, separately and also in combination at their respective concentrations for 48 hours. Subsequently, the combination treatment of IL-6 (at 100pg/ml), IL-10 (at 1000 pg/ml), and TGF-β1 (at 100 pg/ml) will be referred to as combined treatment in this article. These cells were harvested and resuspended in DMEM: F12 at the density of 400,000 cells/ml. The resuspended cells were added into each laminin-coated well and incubated at 37°C. Two hours after the initial incubation, cell supernatant was collected and stored in -20°C for further analyses. Each well was gently washed with phosphate buffer saline (PBS) and Calcein-AM working solution was added into each well and further incubated for 1 h at 37°C. Relative cell attachment on the laminin coated wells were assessed using fluorescence plate reader at an excitation wavelength of ~485 nm and an emission wavelength of ~520 nm. The reaction was performed in triplicates. Untreated TW01 and TW01-LMP1 cells were used as controls.

### ELISA for MMP-3, MMP-9 and VEGF

Quantitation of MMP-3, MMP-9 and VEGF protein in the culture medium was carried out using enzyme-linked immunosorbent assay (ELISA) (Calbiochem, USA) as prescribed in the manufacturer's protocol. Culture medium obtained from cells grown on laminin treated with IL-6 (100 pg/ml), IL-10 (1000 pg/ml), TGF-β1 (100 pg/ml) separately and also in combination at their respective concentration for 48 h, were used. As comparison, culture medium from cells grown without laminin but treated with the same cytokines was used. The reactions were performed in triplicates. Untreated cells were served as control. Absorbance was measured using spectrophotometric plate reader (Tecan, Switzerland) at dual wavelengths of 450/595 nm.

### Caspase-3 Apoptosis Assay

Apoptosis of treated cells were analysed using Caspase 3 Colourimetric Assay kit (Sigma, USA) according to the manufacturer's recommendation. The assay is based on the hydrolysis of the peptide substrate acetyl-Asp-Glu-Val-Asp p-nitroanilide (Ac-DEVD-pNA) by caspase 3, resulting in the release of p-Nitroaniline (pNA) moiety. Apoptosis was induced in TW01 and TW01-LMP1 cells by addition of staurosporine (Sigma, USA) to a final concentration of 1 μg/ml and incubated for 3 h at 37°C in a 5% CO_2 _atmosphere. Cells were then washed with 1 ml PBS, centrifuged and suspended in 1X lysis buffer at a concentration of 10^7 ^cells/100 μl. Cells were incubated on ice for 20 min before centrifugation at 20, 000 × g for 15 min at 4°C. The supernatant was collected and added into a flat bottom 96 wells plate according to the manufacturer's reaction scheme. The plate containing reaction mixture was then covered and placed in a 37°C incubator for 90 min. The reaction was performed in triplicates. Results were analysed using ELISA plate reader (Tecan, Switzerland) at the absorbance of 405 nm.

### Real time quantitative RT-PCR

TW01 and TW01-LMP1 cells were treated with IL-6 (100 pg/ml), IL-10 (1000 pg/ml), TGF-β1 (100 pg/ml) for 48 hours. At the same time, another batch of cells was treated similarly on laminin coated plates. These cells were then harvested and total RNA was extracted using RNeasy Mini Kit (Qiagen, USA).

Real time RT-PCR amplification was performed in an iQ5 Cycler (Biorad, USA). Primers and TaqMan probes for *bmi-1, ngx6 *and the *gapdh *control reference gene were designed and synthesized according to Taqman Gene Expression Assay (assays Hs00958696_g1, Hs00409825_g1, and 4333764F respectively) (Applied Biosystems, USA). PCR reactions were carried out in a total volume of 50 μL, according the manufacturer's instructions. Amplification efficiency was done for all primers with serial dilutions of TW01 RNA (0-times, 10-times and 100-times dilution). The reactions were performed in triplicates.

### Data Analysis

Statistical analysis was performed for the ELISA, laminin adherence test, and apoptosis test results using Student's t-test.

Analysis for real time RT-PCR was performed using the relative quantification ΔΔCt (delta delta threshold cycle) method. Ct values were first collected from the RT-PCR reactions. ΔCt values were then calculated with the formula below:

$$\Delta \text{Ct}={\text{Ct}}_{\text{gene of interest}}–{\text{Ct}}_{\text{housekeeping gene}}$$

Next, ΔΔCt values were calculated according to the formula:

ΔΔCt=ΔCt test−ΔCt reference

Finally, fold change was calculated using the formula:

$$\text{Fold change}={\text{2}}^{-\Delta \Delta \text{Ct}}$$

Fold change refers to the relative fold change of the amount of gene expressed by a particular sample of interest compared to any chosen group of reference.

### Ethics Approval

This study was approved by the International Medical University Joint Committee on Research and Ethics in the year 2008.

## Results

### Expression of LMP1 in transfected TW01 cells

**Figure 1:**
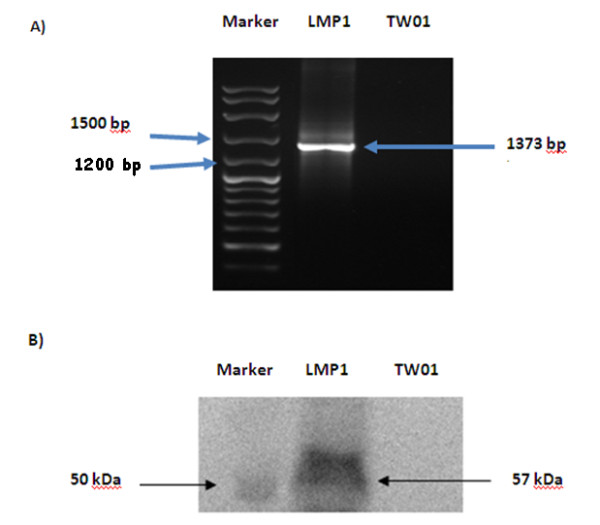
**The overexpression of LMP1 in TW01-LMP1 cells was confirmed by RT-PCR and Western Blotting with specific antibodies**. A) The LMP1 gene was detected from the EBV LMP1-transfected by RT-PCR. B) LMP1 expression was detected as the 57 kDa protein. LMP1 represents TW01-LMP1 cell line while TW01 represents LMP1 negative TW01 cell line. TW01 served as negative control in this result.

Overexpression of LMP1 in LMP1-transfected TW01 cell line was confirmed using RT-PCR and western blot analysis shown in Figures 1A and 1B. The RT-PCR product of LMP1 from the prototype B95.8 Epstein-Barr virus was represented by a band with the fragment size 1373 bp while in western blotting, a band of 57kDa was detected, indicating the expression of the LMP1 protein in transfected TW01 cells.

### MMPs and VEGF production

Cell were treated with IL-6 (100 pg/ml), IL-10 (1000 pg/ml), TGF-β1 (100 pg/ml) separately and also in combination at their respective concentration for 48h and MMP-3, MMP-9 and VEGF produced was quantified using ELISA.

**Figure 2:**
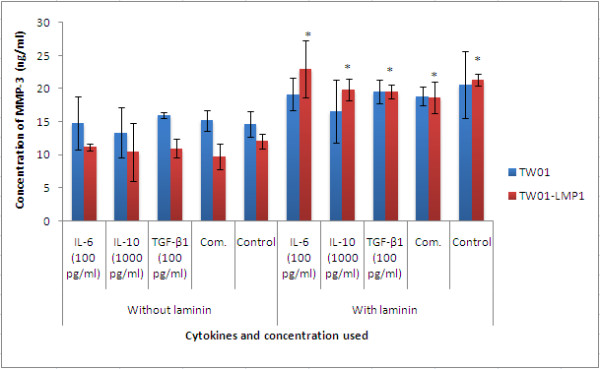
**The expression of MMP-3 in TW01-LMP1 and TW01 cells was measured by quantitative sandwich ELISA**. Each bar represents the mean ± SD value for assays conducted in triplicates. The term 'Comb.' found in the chart represents combined treatment consisting of IL-6 (100 pg/ml), IL-10 (1000 pg/ml), and TGF-β1 (100 pg/ml). The indication '*' represents the significance of p < 0.05.

**Figure 3:**
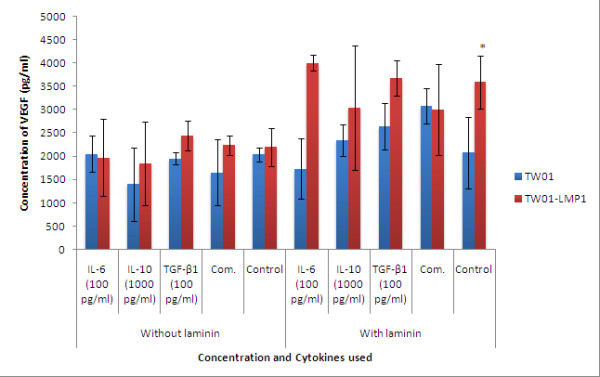
**The expression of VEGF in TW01-LMP1 and TW01 cells was measured by quantitative sandwich ELISA**. Each bar represents the mean ± SD value for assays conducted in triplicates. The term 'Comb.' found in the chart represents combined treatment consisting of IL-6 (100 pg/ml), IL-10 (1000 pg/ml), and TGF-β1 (100 pg/ml). The indication '*' represents the significance of p < 0.05.

**Figure 4:**
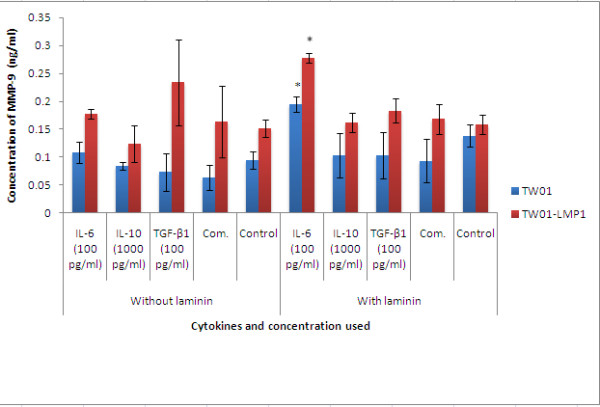
**The expression of MMP-9 in TW01-LMP1 and TW01 cells was measured by quantitative sandwich ELISA**. Each bar represents the mean ± SD value for assays conducted in triplicates. The term 'Comb.' found in the chart represents combined treatment consisting of IL-6 (100 pg/ml), IL-10 (1000 pg/ml), and TGF-β1 (100 pg/ml). The indication '*' represents the significance of p < 0.05.

Treatment with IL-6, IL-10, TGF-β1 individually and in combination had no effect on the expression of MMP-3 and VEGF in TW01 cells (Figures 2 and 3). However, the presence of LMP1 in TW01-transfected cells was found to significantly induce the production of MMP-3 and VEGF in the presence of laminin (both with the p-values < 0.05) IL-6 (100 pg/ml) was found to significantly increase the production of MMP-9 (p < 0.05) in both TW01 and TW01-LMP1 cells when cultured on laminin (Figure 4).

### Resistance to apoptosis

**Figure 5:**
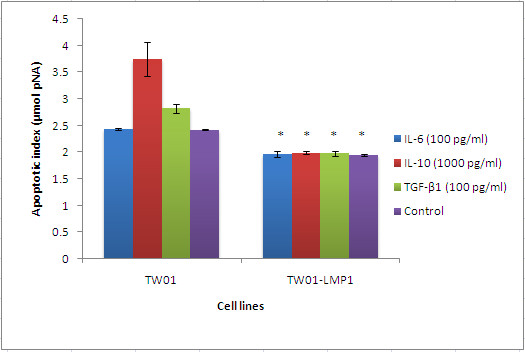
**The apoptotic index of TW01 and TW01-LMP1 cells when subjected to different cytokines**. The level of apoptosis was correlated to the level of hydrolysed p-nitroaniline (pNA) measured at 405 nm. TW01-LMP1 cells were found to have reduced apoptotic index as compared to TW01 cells. Each bar represents the mean ± SD value for assays conducted in triplicates. The term 'Comb.' found in the chart represents combined treatment consisting of IL-6 (100 pg/ml), IL-10 (1000 pg/ml), and TGF-β1 (100 pg/ml). The indication '*' represents the significance of p < 0.05.

Apoptosis was induced in TW01 and TW01-LMP1 cells by addition of staurosporine and was analysed for activation of caspase 3. Interleukin-10 (1000 pg/ml) enhanced apoptosis in TW01 cells but this effect was abolished in TW01 cells expressing the EBV LMP1 (TW01-LMP1) (Figure 5). IL-6, and TGF-β1 had no significant effects on the apoptotic index of TW01 and TW01-LMP1 cells. However, a comparison between TW01 and TW01-LMP1 concludes that the presence of LMP1 alone was sufficient in conferring resistance to apoptosis (p < 0.05) (Figure 5).

### Attachment to laminin

**Figure 6:**
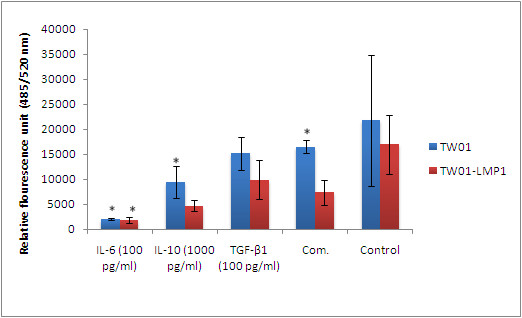
**The attachment of TW01 and TW01-LMP1 cells towards laminin after subjected to the cytokine treatments**. The treatment of IL-6 (100 pg/ml), IL-10 (1000 pg/ml), TGF-β1 (100 pg/ml), and combined treatment consisting of IL-6 (100 pg/ml), IL-10 (1000 pg/ml), and TGF-β1 (100 pg/ml) were subjected on TW01 and TW01-LMP1 cells. Each bar represents the mean ± SD value for assays conducted in triplicates. The term 'Comb.' found in the chart represents combined treatment consisting of IL-6 (100 pg/ml), IL-10 (1000 pg/ml), and TGF-β1 (100 pg/ml). The indication '*' represents the significance of p < 0.05.

The relative cell attachment to laminin was assessed using the Innocyte™ ECM Cell Adhesion Assay, Laminin/Basement Membrane Complex (Calbiochem, USA). Based on cell adherence experiment (Figure 6), it was found that TW01-LMP1 cells had lower adherence towards laminin (p < 0.05) as compared to TW01 cells. When treated with IL-6 (100 pg/ml), IL-10 (1000 pg/ml) and combined treatment, cellular attachment towards laminin was reduced in TW01 cells (p < 0.05). In TW01-LMP1 cells, the treatment of IL-6 at 100 pg.ml significantly reduced the cell adherence towards laminin (p < 0.05). Treatment with TGF-β1 (100 pg/ml) showed no effect on the adherence of both TW01 and TW01-LMP1 cells towards laminin.

### Comparison of *bmi-1 *and *ngx6 *expression in TW01 and TW01-LMP1 cells

**Figure 7:**
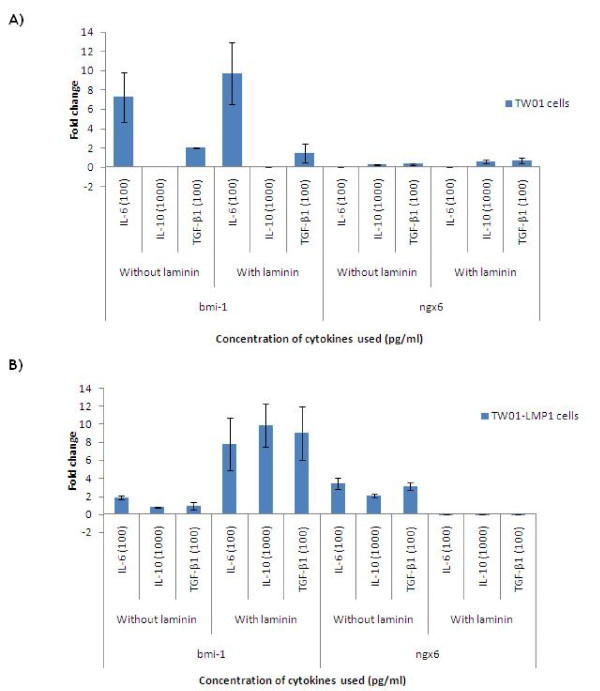
**The fold change of bmi-1 and ngx6 expression in TW01 and TW01-LMP1 cells**. A) Expression of *bmi-1 *and *ngx6 *in treated TW01 cells. B) Expression of *bmi-1 *and *ngx6 *in treated TW01-LMP1 cells. Each bar represents the mean ± SD value for assays conducted in triplicates. The term 'Comb.' found in the chart represents combined treatment consisting of IL-6 (100 pg/ml), IL-10 (1000 pg/ml), and TGF-β1 (100 pg/ml).

Expression of *bmi-1 *and *ngx6 *wsa carried out using real-time RT-PCR amplification with *gapdh *as the reference gene. IL-6 (100 pg/ml) induced the up-regulation of *bmi-1 *gene expression by 7.2 and 9.68-fold, respectively, in TW01 cells that were cultured with and without laminin. Similar observation was also noted in TW01-LMP1 cells cultured in laminin (7.78-fold). IL-10 and TGF-β1 was found to up-regulate the *bmi-1 *expression in TW01-LMP1 cells (9.89-fold and 8.98-fold) cultured with laminin but not in TW01 (Figure 7).

On the contrary, treatment with IL-6 (100 pg/ml) significantly down-regulated the expression of *ngx6 *in TW01 cells that were cultured with (0.00005061-fold) and without (0.0000027-fold) of laminin. Similar observation was also noted in TW01-LMP1 cells cultured in laminin (0.00526-fold). These observations indicated the synergistic relationship between IL-6, EBV LMP1 and laminin in up-regulating the expression of *bmi-1 *oncogene and down-regulating the *ngx6 *tumour suppressor gene.

## Discussion

Although inflammation has long been related to tissue irritation, injury, or infection, it has recently been associated with a wide variety of diseases including cancer. Many studies have linked the relationship between interleukins and growth factors in cancer metastasis. However, no studies have yet been done on the role of IL-6, IL-10 and TGF-β1 in the metastasis of NPC. High levels of MMP-3, MMP-9 and VEGF have all been associated with metastasis in NPC and can therefore serve as endpoint measurement for metastatic disease. It was also discovered that *bmi-1 *oncogene was up-regulated while *ngx6 *tumour suppressor gene was down-regulated during the development of tumours. Expression of *bmi-1 *and *ngx6 *can therefore serves as an indicator for tumour development.

The concentrations of IL-6, IL-10 and TGF-β1 used in this study was selected based on a previous study done by Tan *et al.*, which reported on changes in the levels of IL-6, IL-10 and TGF-β1 in NPC patients before and after treatment [14].

The oncogenic properties possessed by LMP1 are attributed to its ability to elevate anti-apoptotic proteins and growth signals [20]. In this study, LMP1 was found to be able to reduce the apoptosis index. TW01 cells treated with IL-10 and TGF-β1 had increased apoptosis index which indicate increased resistance to apoptosis. However, when LMP1 was expressed, the effect was abolished. This concurs with other studies which showed that LMP1 modulate apoptosis via the NF-κB signaling pathway [21-23]. Shao et al. suggested that LMP1 has enhanced survival and proliferation-related signals despite heavy infliltration by lymphocytes in the tumour cells. Our results agreed with Shao et al. that LMP1 has the ability to prevent apoptosis by reduction in the activity of caspase-3 [24].

VEGF is one of the most pivotal angiogenic factors that is important for invasion and metastasis of tumour [25,26]. LMP1 was found to up-regulate the expression of VEGF in TW01 cells. This is consistent with a study which indicated that VEGF expression was significantly elevated in metastatic NPC tissues as compared to its normal counterpart [27]. Another study also supported our findings by reporting that VEGF has been indicated as a marker of tumour invasion and metastasis in squamous cell carcinoma of the head and neck cancer [28]. The ability of LMP1 in inducing the expression of MMPs in NPC via its CTAR-1 and CTAR-2 regions has been commonly reported [3,24,29-31]. This is in line with our findings that, when cultured on laminin, LMP1-expressing cells had enhanced MMP-3 production. It the therefore suggested that there is possibly an interactive role of laminin and LMP1 in the regulation of MMP-3 expression in NPC cells.

The oncogene, *bmi-1*, was found to be positively correlated with poor prognosis in NPC patients, thus became a valuable marker for the NPC patients [32-34]. In the presence of laminin, the expression of *bmi-1 *gene was significantly up-regulated in LMP1-expressing NPC cells. This coincided with the down-regulation of *ngx6*. The gene *ngx6 *is a type of tumour suppressor and this gene has been recently reported to play a role in cell adhesion modulation in NPC [35].

## Conclusion

We conclude that EBV LMP1, IL-6 and laminin have significant roles in promoting invasion and metastasis in NPC through increased production of MMP-9 in LMP1-transfected TW01 cells; in addition to the up-regulation of *bmi-1 *and down-regulation of *ngx6*.

## Competing interests

The authors declare that they have no competing interests.

## Authors' contributions

TEL designed and coordinated the study, and helped in drafting and reviewing the manuscript. GSY helped in reviewing the manuscript. TEL and AKSB contributed in the application for grant. CMMS carried out the whole study, participated in the study design and drafted the manuscript. All authors read and approved the final manuscript.

## Pre-publication history

The pre-publication history for this paper can be accessed here:

http://www.biomedcentral.com/1471-2407/10/574/prepub
